# PET imaging of soluble epoxide hydrolase in non-human primate brain with [^18^F]FNDP

**DOI:** 10.1186/s13550-020-00657-7

**Published:** 2020-06-22

**Authors:** Yong Du, Il Minn, Catherine Foss, Wojciech G. Lesniak, Feng Hu, Robert F. Dannals, Martin G. Pomper, Andrew G. Horti

**Affiliations:** Division of Nuclear Medicine and Molecular Imaging, The Russell H. Morgan Department of Radiology and Radiological Science, The Johns Hopkins University School of Medicine, 601 North Caroline Street, JHOC 3223, Baltimore, MD 21287 USA

**Keywords:** sEH, Positron emission tomography, Central nervous system, *Papio anubis*, [^18^F]FNDP

## Abstract

**Purpose:**

Soluble epoxide hydrolase (sEH) is a promising candidate positron emission tomography (PET) imaging biomarker altered in various disorders, including vascular cognitive impairment (VCI), Alzheimer’s disease (AD), Parkinson’s disease (PD), stroke, and depression, known to regulate levels of epoxyeicosatrienoic acids (EETs) and play an important role in neurovascular coupling. [^18^F]FNDP, a PET radiotracer for imaging sEH, was evaluated through quantitative PET imaging in the baboon brain, radiometabolite analysis, and radiation dosimetry estimate.

**Methods:**

Baboon [^18^F]FNDP dynamic PET studies were performed at baseline and with blocking doses of the selective sEH inhibitor AR-9281 to evaluate sEH binding specificity. Radiometabolites of [^18^F]FNDP in mice and baboons were measured by high-performance liquid chromatography. Regional brain distribution volume (*V*_*T*_) of [^18^F]FNDP was computed from PET using radiometabolite-corrected arterial input functions. Full body distribution of [^18^F]FNDP was studied in CD-1 mice, and the human effective dose was estimated using OLINDA/EXM software.

**Results:**

[^18^F]FNDP exhibited high and rapid brain uptake in baboons. AR-9281 blocked [^18^F]FNDP uptake dose-dependently with a baseline *V*_*T*_ of 10.9 ± 2.4 mL/mL and a high-dose blocking *V*_*T*_ of 1.0 ± 0.09 mL/mL, indicating substantial binding specificity (91.70 ± 1.74%). The *V*_ND_ was estimated as 0.865 ± 0.066 mL/mL. The estimated occupancy values of AR-9281 were 99.2 ± 1.1% for 1 mg/kg, 88.6 ± 1.3% for 0.1 mg/kg, and 33.8 ± 3.8% for 0.02 mg/kg. Murine biodistribution of [^18^F]FNDP enabled an effective dose estimate for humans (0.032 mSv/MBq). [^18^F]FNDP forms hydrophilic radiometabolites in murine and non-human primate plasma. However, only minute amounts of the radiometabolites entered the animal brain (< 2% in mice).

**Conclusions:**

[^18^F]FNDP is a highly sEH-specific radiotracer that is suitable for quantitative PET imaging in the baboon brain. [^18^F]FNDP holds promise for translation to human subjects.

## Introduction

Soluble epoxide hydrolase (sEH) catalyzes the hydrolysis of epoxyeicosatrienoic acids (EETs) to the corresponding diols with reduced biological activity. EETs play an important role in vasodilation of cerebral blood vessels that accompany neuronal activity, i.e., neurovascular coupling [1–3]. Accordingly, sEH is important in cerebrovascular pathophysiology in the context of mild cognitive impairment, vascular cognitive impairment (VCI), Alzheimer’s disease (AD), stroke, stress, depression, and other conditions [4, 5]. Post-mortem and animal studies have shown increased levels of expression of cerebral sEH in age-related VCI [4], anorexia [6], depression [5, 7], bipolar disorder [5], schizophrenia [5], Parkinson’s disease [5, 8, 9], and dementia with Lewy bodies [5, 10, 11]. Imaging of sEH using positron emission tomography (PET) may shed light on those disorders as well as on normal brain function, particularly with respect to neurovascular coupling.

PET imaging of sEH may also prove useful to study target engagement and other aspects of sEH drug candidates for treatment of pain, inflammation, hypertension, and numerous other conditions [12–15]. Perhaps most significantly, PET imaging of sEH may enhance our understanding of the pathophysiology of vascular disease that occurs in MCI and AD [16]. Combined with existing imaging agents for amyloid and tau, PET imaging of sEH may provide complementary mechanistic information for the noninvasive methods by which to understand the neurobiology of dementia [17, 18].

We have recently developed a potent sEH inhibitor FNDP. Radiolabeled [^18^F]FNDP is the first PET radiotracer for specific imaging of sEH [19]. Our previous studies have demonstrated that [^18^F]FNDP readily entered the mouse [5% injected dose per gram (ID/g) of tissue] and baboon (standardized uptake value, SUV = 4) brain, binding sEH with high specificity in self-blockade experiments (~ 95%).

**Fig. 1:**
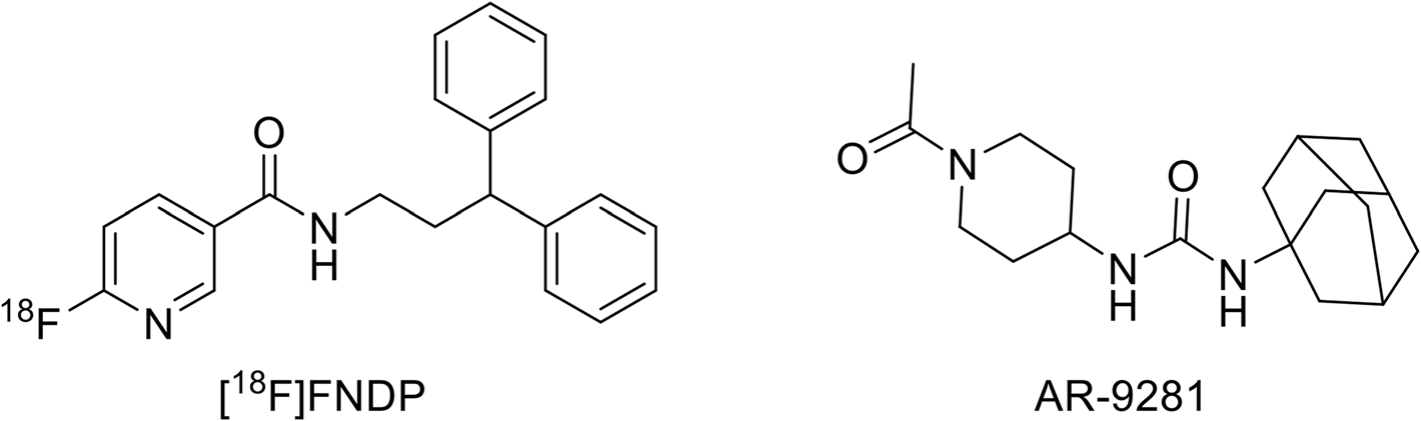
Structures of [^18^F]FNDP and AR-9281

To confirm the very high binding specificity of [^18^F]FNDP to sEH and its promising in vivo imaging properties, we performed extensive baboon PET imaging studies (baseline and dose-escalation blockade with selective sEH inhibitor AR-9281) (Fig. 1), quantification of [^18^F]FNDP radiometabolites in murine and baboon plasma and brain, and whole body biodistribution in mice to provide estimates of radiation burden to the human body.

## Materials and methods

All experimental animal protocols were performed in accordance with relevant federal and state laws and institutional guidelines and approved by the Animal Care and Use Committee of the Johns Hopkins Medical Institutions.

### Chemicals

AR-9281, a potent and selective sEH inhibitor [14, 20–23] tested in Phase 2 human trial (ClinicalTrials.gov identifier: NCT00847899), was synthesized in-house as described previously [22]. [^18^F]FNDP was radiolabeled as described previously [19, 24].

### Baboon PET imaging

PET images were acquired using a CPS/CTI high-resolution research tomograph (HRRT), which has an axial resolution (FWHM) of 2.4 mm and in-plane resolution of 2.4–2.8 mm [25]. The baseline studies were performed on three male baboons (*Papio anubis*) weighing 22–26 kg and the dose-escalation blocking scans with AR-9281 were performed on one of these animals. These blocking studies were performed 27 to 43 days apart from each other to ensure full animal recovery. The animals had no food for 12 h prior to each PET study. Anesthesia was induced with intramuscular ketamine (7.5–10 mg/kg) and maintained with a continuous intravenous infusion of propofol at 0.3–0.4 mg/kg/min throughout the PET experiment. One venous catheter was inserted for the radioligand injection, and one arterial catheter was inserted to obtain arterial blood samples. Baboons were intubated to facilitate respiration, and circulatory volume was maintained by constant infusion of isotonic saline. Physiological vital signs including heart rate, blood pressure, electrocardiogram, and oxygen saturation were monitored continuously throughout the study.

The animals were positioned in the PET scanner with the head immobilized using a thermoplastic mask. A 6-min transmission scan was acquired using a rotating ^137^Cesium source for generating attenuation maps. The 90-min dynamic PET acquisition was then started in three-dimensional list mode simultaneously with an intravenous bolus injection of [^18^F]FNDP. For the three blocking studies in the same animal, AR-9281 was given subcutaneously 1 h before the intravenous bolus injection of [^18^F]FNDP and the start of the dynamic scan. Table 1 provides experimental details of the PET studies.

**Table 1:** Experimental information for [^18^F]FNDP PET scans (baseline and blocking) in baboons

Animal	Study	Days after baseline scan	[^18^F]FNDP dose (MBq)	Specific activity, TBq/μmol (Ci/μmol)	FNDP carrier dose, (μg/kg)	AR-9281 dose (mg/kg)
Baboon 1	Baseline	-	314.13	1.30 (35.2)	3.2 × 10^-3^	0
Baboon 2	Baseline	-	247.90	2.63 (71.2)	1.3 × 10^-3^	0
Baboon 3	Baseline	-	295.26	3.65 (98.6)	1.1 × 10^-3^	0
	Blocking 1	27	283.42	2.87 (77.5)	1.3 × 10^-3^	1.0
	Blocking 2	69	294.89	2.96 (79.9)	1.3 × 10^-3^	0.1
	Blocking 3	112	284.16	2.13 (57.5)	1.8 × 10^-3^	0.02

### Input function and radiometabolite analysis

Measurement of the arterial plasma input function was conducted through collection of ~ 40 blood samples over the course of the 90-min dynamic PET study. Blood samples were obtained via the arterial catheter at continually prolonged intervals, as rapidly (< 5 s) as possible for the first 90 s, with samples acquired at increasingly longer intervals thereafter. Samples were then centrifuged at 1200 × *g*, and the plasma radioactivity was measured with a cross-calibrated PerkinElmer Wizard 2480 automatic gamma counter.

Select plasma samples (0, 5, 10, 20, 30, 60, and 90 min) were analyzed for radiometabolites using high-performance liquid chromatography (HPLC). The modified column-switching HPLC method was used [26]. The HPLC system consisting of a 1260 infinity quaternary pump, a 1260 infinity column compartment module, a 1260 infinity UV detector, and a Raytest GABI Star radiation detector, was operated with OpenLab CDS EZChrom (A.01.04) software. A 0.4–1.5-mL sample of plasma was loaded into a 2-mL Rheodyne injector loop and initially directed to the capture column (packed with Phenomenex Strata-X 33 μm polymeric reversed phase sorbent) and both detectors with 1% acetonitrile and 99% water mobile phase at 2 mL/min. After 1 min of isocratic elution, analytical mobile phase composed of 65% acetonitrile and 35% water with 0.06 M trimethylamine (at pH = 7.2, adjusted with phosphoric acid) was applied to direct non-polar compounds trapped on the capture column to an analytical column (Gemini C18(2), 10 μm, 4.6 × 250 mm) and detectors at 2 mL/min. The HPLC system was standardized for mass using non-radioactive FNDP prior to analysis of the blood plasma samples, which were spiked with 5 μL of non-radioactive FNDP at a concentration of 1 mg/mL.

### Murine radiometabolite analysis

Male CD-1 mice (25–26 g) were injected via the lateral tail vein with 37 MBq of high specific activity (1.1 TBq/μmol or 29.7 Ci/μmol) [^18^F]FNDP. The mice were sacrificed by cervical dislocation at 10 and 30 min after injection, and a terminal blood sample was obtained. The mouse brains were rapidly removed, homogenized in 2 mL of ACN:H_2_O 50%–50% (vol) with 0.06 M ammonium formate to extract parent tracer and radiometabolites, centrifuged at 20,000 *g* at 4 °C. Resulting supernatants were analyzed by reverse-phase (RP)-HPLC.

### Dynamic PET data analysis

The 90 min of PET data were binned into 30 frames: four 15-s, four 30-s, three 1-min, two 2-min, five 4 -min, and 12 5-min frames. Images were reconstructed using the 3D iterative ordered subset expectation maximization (OS-EM) algorithm (with six iterations and 16 subsets) with correction for radioactive decay, deadtime, attenuation, scatter, and randoms [27]. The reconstructed image space consisted of cubic voxels, each 1.22 mm^3^ in size, and spanning dimensions of 31 cm × 31 cm (transaxially) and 25 cm (axially).

The software package PMOD (v3.7, PMOD Technologies Ltd, Zurich, Switzerland) was used for the image processing and subsequent kinetic analysis. The previously acquired T1-weighted brain MRI images for the baboon were co-registered to the reconstructed dynamic PET images acquired in this study. Through manually matching the co-registered MRI to the INIA19 Template and NeuroMaps Atlas for Primate Brain Image Parcellation and Spatial Normalization [28], 13 representative baboon brain volumes of interest (VOIs) were defined, including frontal and temporal gyri, thalamus, hippocampus, caudate, putamen, amygdala, globus pallidus, insula, hypothalamus, cerebellum, corpus callosum, and white matter. Regional time activity curves (TACs) were then generated for both baseline and blocking PET scans using those VOIs.

Next, kinetic modeling was performed based on the TACs and the metabolite-corrected arterial plasma input functions. For brain uptake, the primary outcome measure was the regional brain distribution volume (*V*_*T*_) of [^18^F]FNDP, defined as concentration of the radiotracer in regional tissue relative to that in blood at equilibrium [29]. We evaluated which compartmental models (e.g., two-tissue-three-compartment (2TCM) or one-tissue-two-compartment (1TCM)) best described the [^18^F]FNDP PET data, using statistical model selection criteria, such as the Akaike information criterion [30]. For comparison, the Logan graphical method (t* = 30 min) was also used to compute regional *V*_*T*_, as well as generating voxel-wise parametric images of *V*_*T*_ [31].

For blocking studies, occupancy of sEH by FNDP was calculated using regional *V*_*T*_ values as

Eq 1 $$ \mathrm{Occupancy}=\frac{\Delta {V}_T}{V_T^{\mathrm{Baseline}}-{V}_{\mathrm{ND}}}, $$

where *V*_ND_, the distribution volume of non-displaceable radioligand, was obtained from the Lassen plot, i.e., using a scatterplot of Δ*V*_*T*_ (i.e., baseline *V*_*T*_ minus post-blocking *V*_*T*_) versus baseline *V*_*T*_ of all regions [32, 33]. The assumption for this method was that regions showed similar occupancies (i.e., a linear model was supported). When that condition was satisfied, *V*_ND_ was given as the intercept of the regression line with the *X*-axis. The non-displaceable binding potential (BP_ND_) was computed as *V*_*T*_ /*V*_ND_ − 1.

### Full-body radiation dosimetry for [^18^F]FNDP in mice

Radiation dosimetry for [^18^F]FNDP was studied in CD-1 mice (males, 23–27 g) following the procedure of reference [34]. A solution of 3.7 MBq (0.1 mCi) [^18^F]FNDP in 0.2 mL of saline was injected into the lateral tail vein, and groups of mice (*n* = 3 per time point) were euthanized at 15, 30, 60, 120, and 180 min after injection. The organs (lungs, heart, kidneys, liver, spleen, intestine, stomach, and brain) were quickly dissected on ice. Left femur and samples of muscle and blood were also collected. The organs were weighed, and the organ radioactivity was measured with an automated gamma counter (LKB Wallac 1282 CompuGamma CS Universal Gamma Counter). All measurements were corrected for decay. The percent injected dose per organ (%ID/organ) was calculated.

Further analysis was performed commercially (RADAR, Inc, Nashville, TN). In brief, the values of percent of injected activity per organ were fit using SAAM II software [35]. Time integrals of activity were entered into the OLINDA/EXM software [36], using the adult male model. Activity observed in the gastrointestinal (GI) tract was assumed to enter the small intestine and was treated by the standard GI kinetic model. Accumulation of activity was observed in the urinary bladder; activity in the whole body, minus that in the GI tract, was fitted; and the observed biological half time was used in the standard voiding urinary bladder model. The number of disintegrations in the ‘remainder of body’ was assumed to be equal to 100% of the activity administered integrated to total decay of ^18^F, minus the disintegrations in other body organs.

## Results

### Baboon PET studies

#### Plasma and tissue time−activity curves

**Fig. 2:**
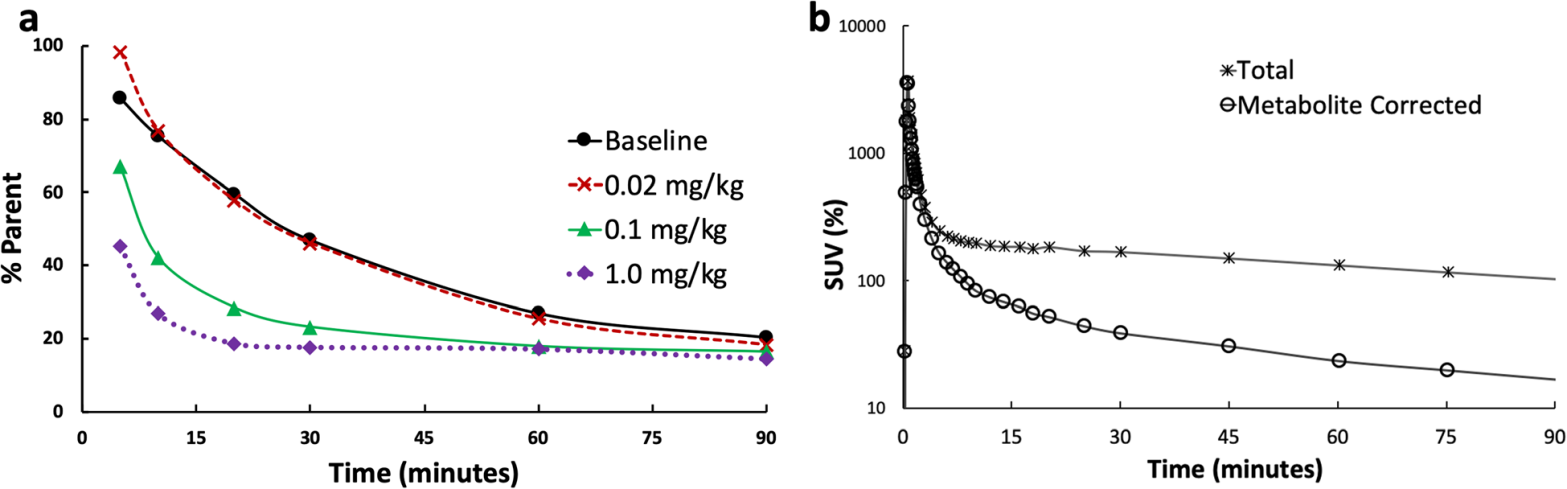
Radioactive metabolites from four scans (baseline and blocking studies on Baboon3) (**a**), and plasma activity with and without metabolite correction from blocking scan with 0.1 mg/kg dose (**b**)

In plasma, the amount of [^18^F]FNDP gradually decreased after injection while radiometabolites gradually increased (Fig 2a). The radiometabolism rate was faster in the blocking scans with higher doses (i.e., 1 mg/kg and 0.1 mg/kg) of AR-9281 than those in the baseline scan and low-dose (0.02 mg/kg) blocking scan. Figure 2b shows the plasma activity with and without metabolite correction for the 0.1 mg/kg blocking study.

**Fig. 3:**
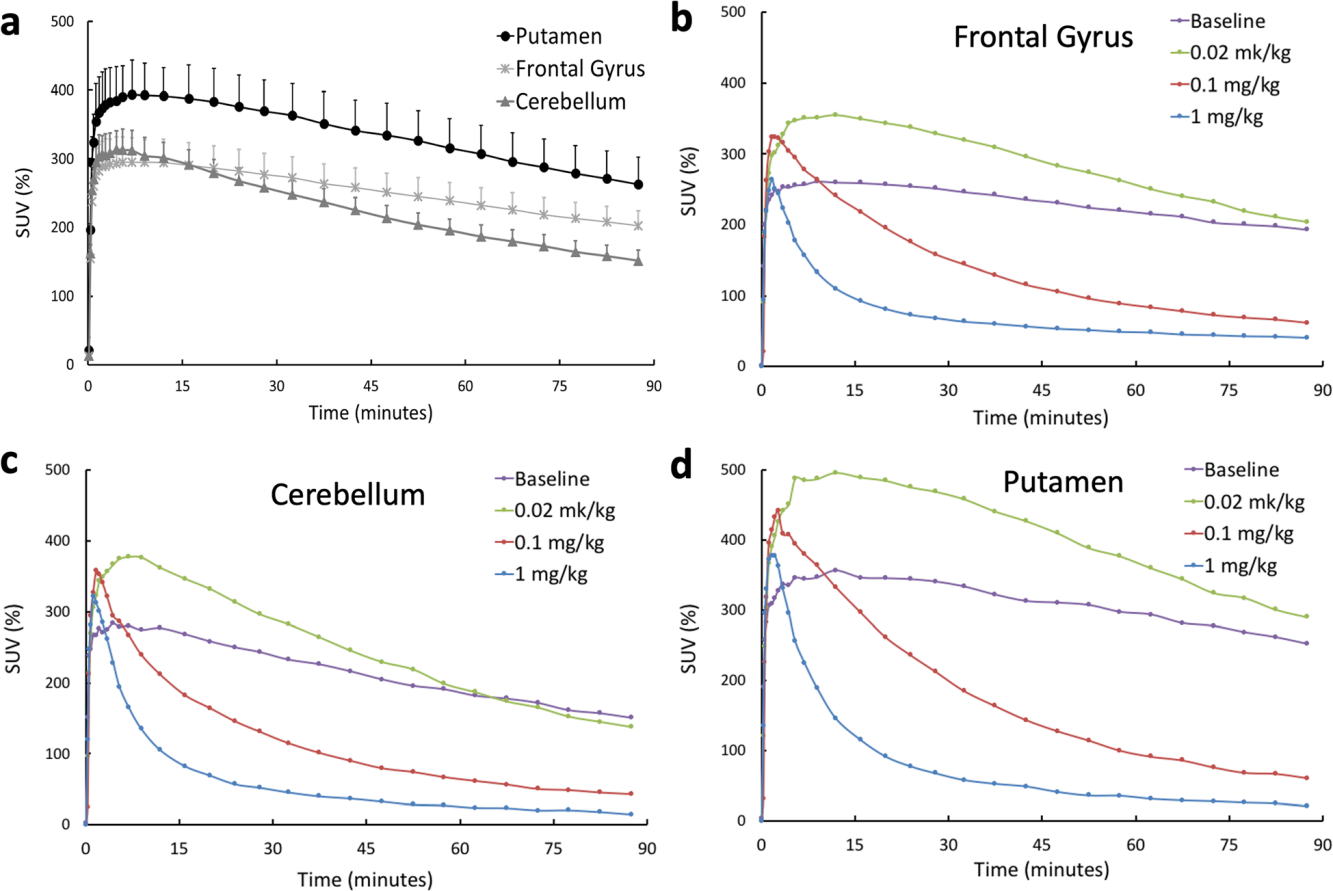
**a** The baseline time–activity curves (TACs) of selected brain regions showing mean and standard deviation from baseline scans of three animals. **b** TACs of frontal gyrus from baseline and three blocking scans (Baboon3). **c** TACs of cerebellum from baseline and three blocking scans (Baboon3). **d** TACs of putamen from baseline and three blocking scans (Baboon3)

Figure 3 shows the SUV time−activity curves (TACs) of selected brain regions from baseline and blocking scans. The TACs in Fig. 3a present the mean values and standard deviations computed from baseline scans in three baboons. For all regions at baseline, TACs peaked before 15 min post-injection, and then gradually decreased with time. The results from all three animals were very similar to each other as demonstrated by the small standard deviation.

For blocking scans in Baboon3, when high doses (0.1 mg/kg and 1 mg/kg) AR-9281 were used for blocking, the TACs peaked earlier and decreased rapidly after peaking for all the brain regions, indicating high occupancy of the tracer binding site by AR-9281. When a blocking dose of 0.02 mg/kg was used, the peaks of the regional TACs were higher than those of the baseline, while decreasing at a faster rate than in the baseline study.

#### Tissue compartmental model

Regional *V*_*T*_ and *K*_1_ values given by 1TCM, 2TCM and *V*_*T*_ values from the Logan graphical method are listed in Table 2 for the baseline scan. Results show the mean and standard deviation from the three baboons used in this study. The *V*_*T*_ values from the 1TCM were smaller than those from the 2TCM and the Logan method. Supplemental Fig S1 compares model-fitted TACs between the 1TCM and 2TCM for Baboon3. The results from the 2TCM matched the measured results while some discrepancies were seen for the 1TCM results. The AIC (Akaike information criteria) values computed from 2TCM were always smaller than the corresponding AIC values computed from 1TCM. A multivariate analysis of variance (MANOVA) was performed where the tissue compartmental model method was an independent variable and the AIC data from each animal was dependent variable. The resulting *P* < 0.005 is significant after Bonferroni correction for multiple comparison (0.05/3 = 0.0133), indicating that the 2TCM is a preferred model for studying [^18^F]FNDP uptake in the brain. Table 2 also shows that there is very good agreement between Logan estimated *V*_*T*_ and *V*_*T*_ estimated from the 2TCM (Pearson’s *r* = 0.99, *P* < 0.001). Therefore, the Logan method was used to compute *V*_*T*_ parametric images and to compare the baseline and blocking scans.

**Table 2:** Estimated parameters from baseline scan of three baboons using different compartmental models

	2TCM		1TCM		Logan
	*V*_*T*_ (mL/mL)	*K*_1_ (mL/cm^3^/min)	*V*_T_ (mL/mL)	*K*_1_ (mL/cm^3^/min)	*V*_T_ (mL/mL)
Thalamus	7.90 ± 0.74	0.17 ± 0.03	7.42 ± 0.36	0.16 ± 0.03	7.87 ± 0.55
Hypothalamus	8.32 ± 0.57	0.15 ± 0.03	7.77 ± 0.35	0.14 ± 0.03	8.08 ± 0.44
Hippocampus	11.47 ± 2.61	0.15 ± 0.04	10.27 ± 1.55	0.14 ± 0.03	10.97 ± 1.96
Temporal gyrus	9.77 ± 1.02	0.17 ± 0.03	9.05 ± 0.41	0.16 ± 0.03	9.52 ± 0.57
Frontal gyrus	10.50 ± 1.01	0.18 ± 0.04	9.58 ± 0.60	0.16 ± 0.04	10.29 ± 0.91
Corpus callosum	9.28 ± 0.90	0.17 ± 0.03	8.32 ± 0.91	0.15 ± 0.03	9.06 ± 0.99
Globus pallidus	9.74 ± 1.02	0.19 ± 0.03	9.07 ± 0.66	0.17 ± 0.03	9.44 ± 0.92
White matter	9.29 ± 1.08	0.18 ± 0.04	8.43 ± 0.89	0.17 ± 0.04	9.00 ± 1.03
Occipital cortex	7.82 ± 1.82	0.18 ± 0.06	7.39 ± 1.56	0.17 ± 0.06	7.67 ± 1.63
Cerebellum	7.66 ± 0.72	0.19 ± 0.03	7.33 ± 0.45	0.18 ± 0.03	7.63 ± 0.58
Amygdala	12.49 ± 2.47	0.14 ± 0.03	10.51 ± 0.81	0.14 ± 0.03	11.98 ± 1.63
Putamen	13.80 ± 2.27	0.23 ± 0.05	12.87 ± 1.54	0.22 ± 0.05	13.32 ± 1.81
Caudate	13.90 ± 2.28	0.21 ± 0.05	12.95 ± 1.44	0.21 ± 0.05	13.35 ± 1.94
Insula	13.76 ± 1.42	0.22 ± 0.05	12.13 ± 1.08	0.20 ± 0.05	12.82 ± 1.32
AIC	− 38.96 ± 33.09		− 1.65 ± 12.24		-

*The results show mean and standard deviation computed from 3 baboons’ parameters.

**Fig. 4:**
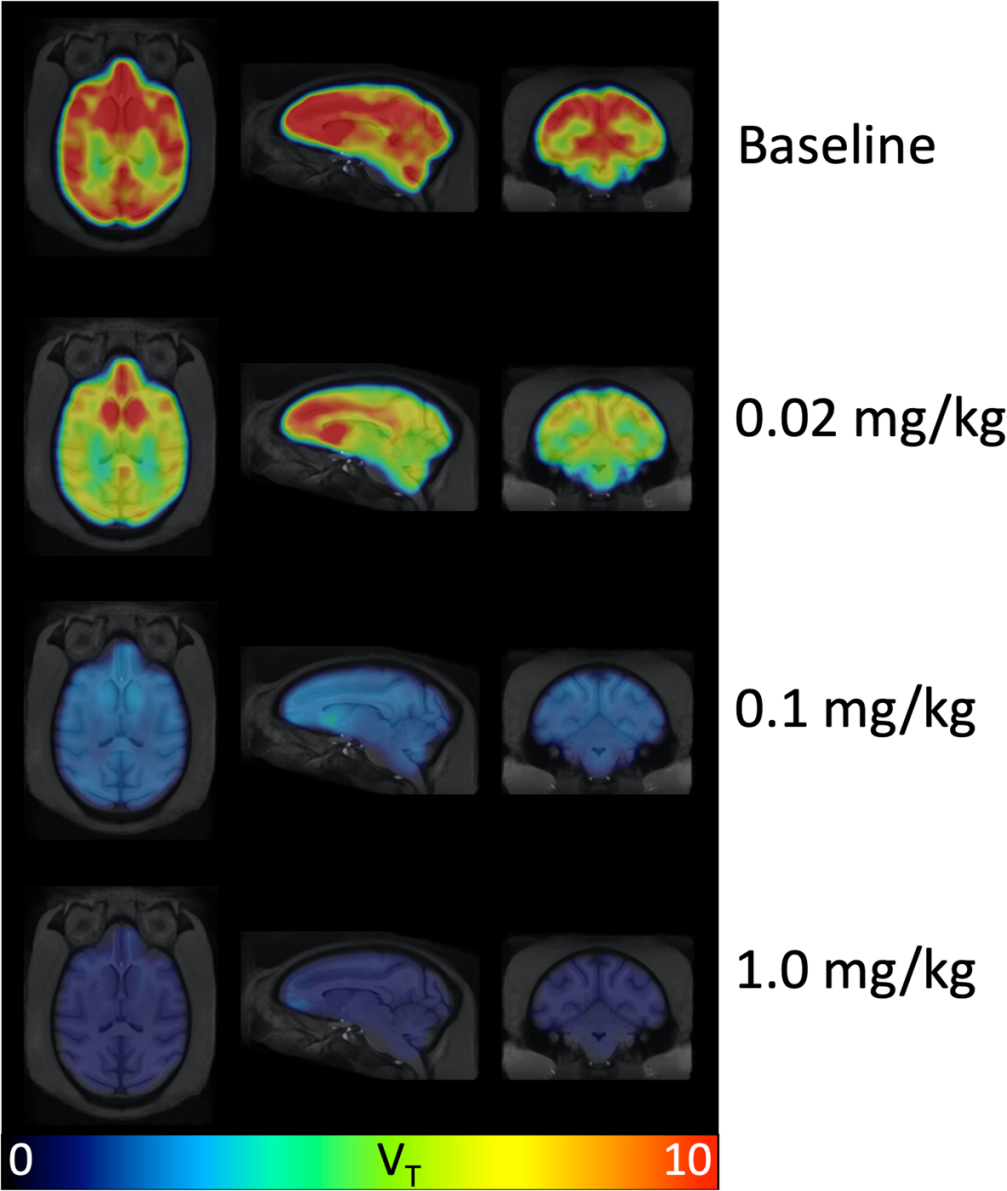
*V*_*T*_ parametric images from baseline and three blocking scans with AR-9281 in Baboon3. The Logan graphic method was used to generate the *V*_*T*_ map

**Fig. 5:**
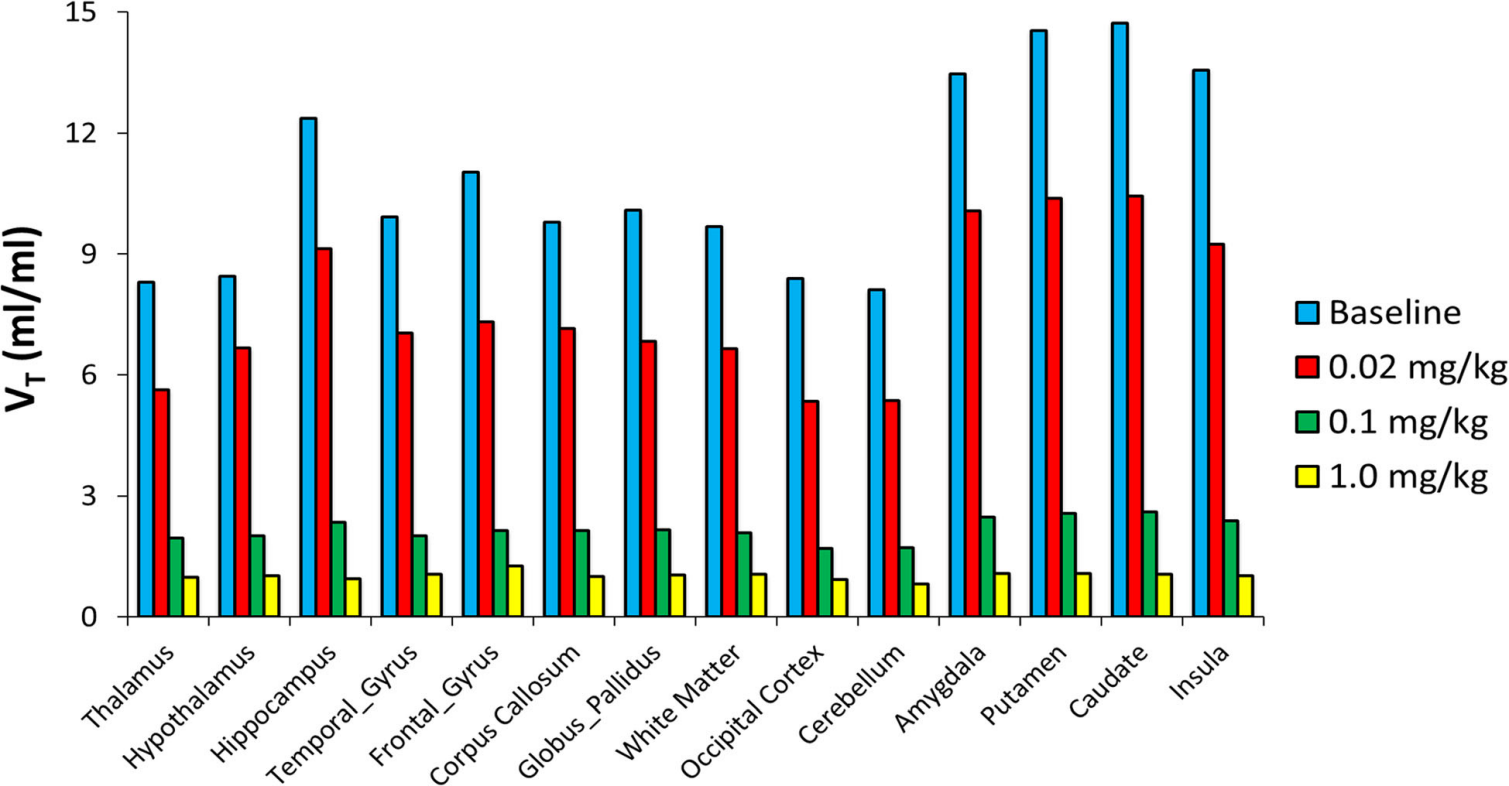
The regional *V*_*T*_ from baseline scan and three blocking scans with AR-9281 estimated from the Logon graphic method for Baboon3

Figure 4 compares the *V*_*T*_ parametric images from baseline and three blocking scans computed using the Logan method for Baboon3. The regional *V*_*T*_ values are shown in Fig. 5 for Baboon3. At baseline, the *V*_*T*_ values had a large variation among different brain regions with the smallest value in the cerebellum and the highest value in the striatum, respectively. Blocking with AR-9281 reduced uptake in all brain regions, with the higher doses causing more significant reduction demonstrating high sEH specific binding (see below).

From the 2TCM the estimated *K*_1_ values were 0.151 ± 0.021 mL/cm^3^/min for the baseline scan, 0.166 ± 0.032 mL/cm^3^/min for the 1-mg/kg blocking scan, 0.179 ± 0.026 mL/cm^3^/min for the 0.1-mg/kg blocking scan, and 0.170 ± 0.021 mL/cm^3^/min for the 0.02-mg/kg blocking scan, respectively, for Baboon3. The paired Student’s *t*-test indicated that there were no significant differences in *K*_1_ values estimated from the three blocking scans. However, there is a significant difference between *K*_1_ from baseline and blocking scans, with the 0.1-mg/kg dose (*P* = 0.003) and the 0.02-mg/kg dose (*P* = 0.022).

**Fig. 6:**
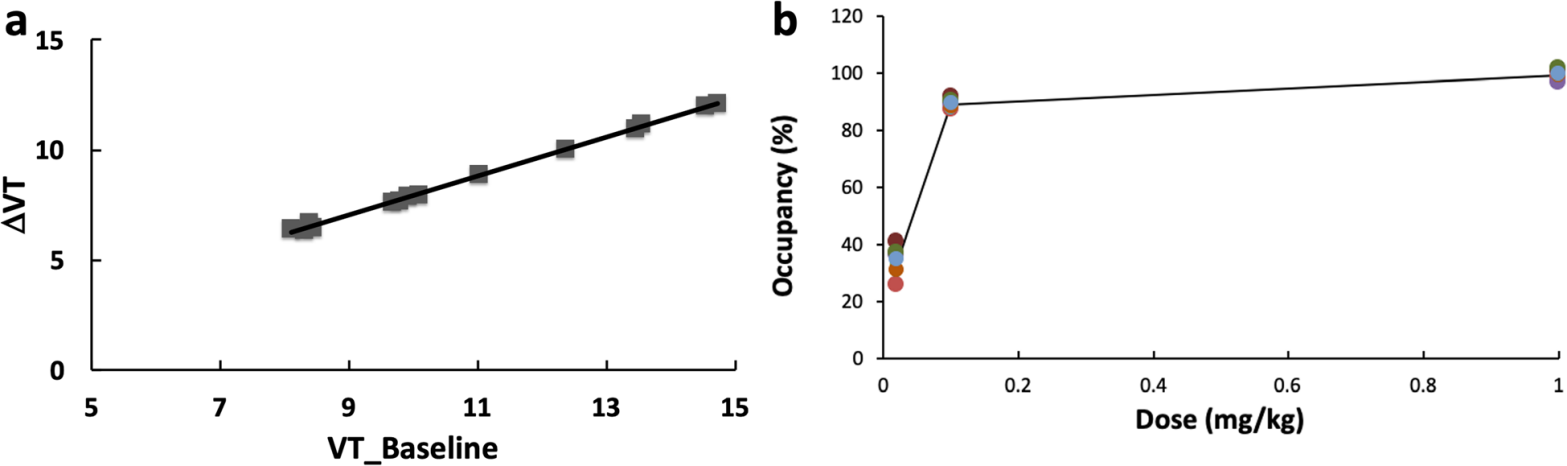
Scatter plots of Δ*V*_*T*_ vs baseline *V*_*T*_ from a blocking study with 0.1 mg/kg dose (**a**), and the computed occupancy as a function of the blocking dose for all brain regions (**b**)

The scatter plot of Δ*V*_*T*_ versus baseline *V*_*T*_ is shown in Fig. 6a for the blocking study with 0.1 mg/kg dose. The results fit a linear model well, with *R*^2^ = 0.998, 0.998, and 0.848 for the blocking dose of 1 mg/kg, 0.1 mg/kg, and 0.02 mg/kg, respectively. Based on the scatter plot, the mean *V*_ND_ was estimated to be 0.865 ± 0.066 mL/mL. This value is much lower than the *V*_*T*_ (from 7.63 to 13.35 in Table 2), indicating lack of an sEH-free region in the baboon brain and low nonspecific binding of [^18^F]FNDP across the whole brain. The BP_ND_ was calculated (*V*_*T*_/*V*_ND_ − 1) to be ranging from 8.37 to 16.02 (unitless), and the specific binding was estimated to be 91.70 ± 1.74%.

A plot of occupancy versus AR-9281 dose is shown in Fig. 6b for all brain regions studied. The estimated occupancy was 99.2 ± 1.1% for the 1-mg/kg dose, 88.6 ± 1.3% for the 0.1-mg/kg dose, and 33.8 ± 3.8% for the 0.02-mg/kg dose, respectively. Because there were only three blocking doses, the ED_50_ was not estimated.

**Fig. 7:**
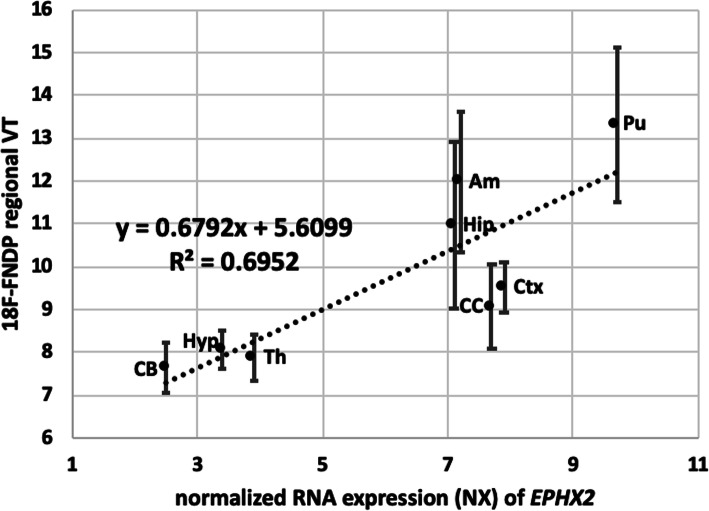
Correlation of baboon regional *V*_*T*_ values (Logan) for [^18^F]FNDP vs. normalized mRNA levels (NX) for *EPHX2* (HPA mouse brain RNA-seq dataset) [37, 38]. Abbreviations: *Pu* putamen, *Ctx* cortex, *CC* corpus callosum, *Am* amygdala, *Hip* hippocampus, *Th* thalamus, *Hyp* hypothalamus, *CB* cerebellum

Further confirmation that [^18^F]FNDP specifically targets sEH is that the regional distribution of [^18^F]FNDP in the non-human primate brain agrees with previously published sEH regional expression. The *EPHX2* gene encodes sEH. Regional brain distribution of *EPHX2* is available from the online protein atlas [37, 38]. The regional brain uptake of [^18^F]FNDP in non-human primate correlates well with mRNA levels of *EPHX2* (Fig. 7). This correlation supports the specific binding of [^18^F]FNDP at sEH with the understanding that the correlating tracer binding with protein levels of sEH rather than mRNA levels would be more relevant.

### Metabolism of [^18^F]FNDP in mouse and baboon

**Fig. 8:**
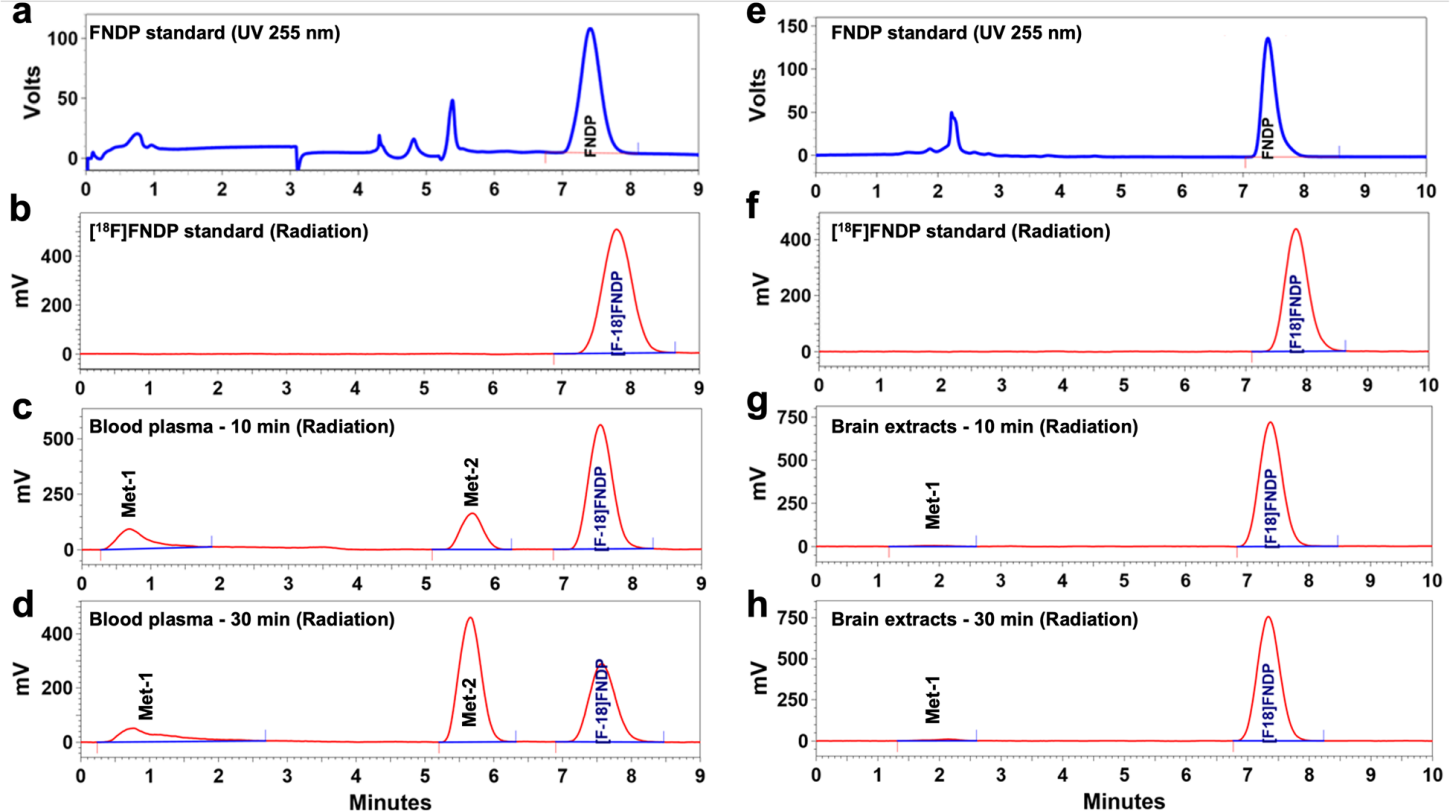
Metabolism of [^18^F]FNDP in mouse blood and brain. Representative HPLC chromatograms recorded for non-radioactive FNDP standard (blue), [^18^F]FNDP standard (red) and blood plasma and brain extract samples obtained from mice injected with 1 mCi of [^18^F]FNDP and sacrificed at 10 and 30 min after radiotracer injection (red). The offset (~ 0.5 min) between UV and radiation peak retention times is because the UV and radiation detectors are connected in series. **a**, **b**, **c**, and **d** Plasma samples are analyzed with inclusion of a capture column. **e**, **f**, **g** and **h** Brain extracts are analyzed with no capture column. The study demonstrated presence of two major radiometabolites (Met-1 and Met-2) in the mouse plasma, but only a small amount of radiometabolite Met-1 entered the mouse brain

HPLC radiometabolite analysis of blood samples from CD-1 mice and baboons showed that the parent compound, [^18^F]FNDP (retention time = 7.8 min), was metabolized to two major radiolabeled hydrophilic species (Met-1, retention time = 1–2 min; Met-2, retention time = 5.7 min) (Fig. 8). The combined radiometabolites in the plasma reached similar levels of about 50% of total activity at 30 min after administration of [^18^F]FNDP in both species, whereas at 90 min in baboon blood, the combined radiometabolites were ~ 78% of total activity. The radiometabolites do not significantly penetrate the blood–brain barrier (BBB) in mice. A low amount of Met-1 entered the mouse brain (~ 1.7% at 30 min), whereas Met-2 was not detectable in the mouse brain, with more than 98% of unchanged parent present in the brain. See details in Table 3.

**Table 3:** HPLC analysis of radiolabeled metabolites of [^18^F]FNDP in mouse blood and brain and baboon blood

	Met-1, %	Met-2, %	Parent [^18^F]FNDP, %
Mouse blood (10 min)	11.2 ± 4.8	25.4 ± 7.2	63.4 ± 4.4
Mouse blood (30 min)	10.1 ± 0.9	39.8 ± 9.8	50.1 ± 10.5
Mouse brain (10 min)	0.8 ± 0.3	Nondetectable	99.2 ± 0.3
Mouse brain (30 min)	1.7 ± 0.3	Nondetectable	98.3 ± 0.3
Baboon blood (10 min)	7.2 ± 1.9	15.8 ± 2.4	77.0 ± 3.6
Baboon blood (30 min)	16.7 ± 6.5	36.9 ± 3.1	46.5 ± 6.4
Baboon blood (60 min)	22.2 ± 9.3	49.5 ± 7.7	28.3 ± 9.3
Baboon blood (90 min)	22.3 ± 8.0	56.0 ± 7.1	21.8 ± 7.0

### Whole-body human radiation dosimetry estimates

The whole-body [^18^F]FNDP distribution data in mice are shown in Supplemental Tables S1&S2. The mouse distribution results were then extrapolated to humans (adult male model) as shown in Table 4. The estimated effective dose was 0.0317 mSv/MBq (0.0117 rem/mCi). The radiation dose of [^18^F]FNDP to the bladder, ULI (upper large intestine) wall, small intestine, kidneys, and liver were higher than for the other organs.

**Table 4:** Radiation dosimetry estimates for human (adult male)

	Estimated dose	
Target organ	mSv/MBq	rem/mCi
Adrenals Brain Breasts Gallbladder wall LLI (lower large intestine) wall Small intestine Stomach wall ULI (upper large intestine) wall Heart wall Kidneys Liver Lungs Muscle Ovaries Pancreas Red marrow Osteogenic cells Skin Spleen Testes Thymus Thyroid Urinary bladder wall Uterus Total body	1.45E–02 5.76E–03 6.40E–03 2.68E–02 5.86E–02 1.41E–01 1.51E–02 1.59E–01 1.58E–02 8.92E–02 4.16E–02 8.46E–03 1.11E–02 3.36E–02 1.54E–02 1.25E–02 1.29E–02 6.83E–03 1.23E–02 1.58E–02 7.55E–03 6.78E–03 1.66E–01 3.31E–02 1.38E–02	5.37E–02 2.13E–02 2.37E–02 9.92E–02 2.17E–01 5.22E–01 5.60E–02 5.88E–01 5.86E–02 3.30E–01 1.54E–01 3.13E–02 4.10E–02 1.24E–01 5.70E–02 4.61E–02 4.76E–02 2.53E–02 4.56E–02 5.83E–02 2.79E–02 2.51E–02 6.13E–01 1.22E–01 5.11E–02
Effective dose	3.17E–02	1.17E–01

## Discussion

sEH is a bifunctional enzyme with carboxy-terminal hydrolase and amino-terminal lipid phosphatase activities [39]. It is expressed by many cell types in the brain and cerebral blood vessels, including neurons, astrocytes, endothelial cells, and vascular smooth cells [2, 3]. It serves to convert EETs to the corresponding diols within the arachidonic acid cascade. sEH plays a significant role in cerebral blood flow autoregulation and neurovascular coupling by regulating the availability of anti-inflammatory EETs in cerebral vasculature [4, 40]. sEH has been shown to be upregulated in a variety of neuropsychiatric conditions with a putative inflammatory component, including major depression, bipolar disorder, and schizophrenia [11]. There is also evidence showing that inhibition of sEH reduces neuronal damage following acute (stroke, hemorrhage) [14] and chronic neurologic insults (Alzheimer’s, Parkinson’s, and dementia) [41]. sEH is actively pursued both as a biomarker and a drug target in Parkinson’s disease, where it tracks with aggregation of α-synuclein and phosphorylated α-synuclein in the striatum [8]. Elevations in protein levels of sEH are on the order of the decreases seen in dopamine transporter (DAT) in Parkinson’s disease [8]. Notably, DAT is the target of a widely used clinical imaging study [42]. Accordingly, non-invasive [^18^F]FNDP PET imaging of sEH may facilitate mechanistic understanding of those diseases by providing information of in vivo availability and distribution of sEH in the human brain. That will enable us to understand better the pathophysiology of vascular components of a variety of disorders including stroke, Alzheimer’s dementia, and Parkinson’s disease. In addition, pharmacological inhibition of sEH is under clinical investigation for treatment of hypertension, atherosclerosis, inflammation, and immunological disorders [12, 43]. PET imaging of sEH can also be used for dose selection, occupancy studies, and therapeutic monitoring in patients undergoing treatment with such sEH inhibitors.

### Baseline [^18^F]FNDP PET in non-human primate brain

The baboon baseline PET scans in three animals demonstrated high uptake of [^18^F]FNDP in most brain regions, in agreement with our previous results [19]. The uptake can be accurately and robustly modeled with a 2TCM. We chose to use the Logan graphical method for analysis because it provided *V*_*T*_ values that agreed well with the 2TCM estimates.

### Blockade of [^18^F]FNDP with AR-9281 in non-human primate brain

Previous [^18^F]FNDP blocking studies have been performed with its analog, nor-fluoro-FNDP [19]. Currently, there are many structurally distinct sEH inhibitors, but a conventional pharmacophore model for sEH inhibitors is not yet available [44, 45]. Many sEH inhibitors are urea derivatives, whereas several of the latest compounds, including FNDP, are not urea-based. Therefore, to prove that the very high specific binding of [^18^F]FNDP [19] is truly mediated by sEH, we demonstrated that [^18^F]FNDP binding can be blocked by the structurally different sEH inhibitor, AR-9281 (Fig. 1). AR-9281 is a highly selective and potent sEH inhibitor (IC_50_ = 8 nM) that was previously studied in Phase 2 clinical trials. It is a BBB-permeable drug [14, 20–23]. As shown in Figs. 3, 4, and 5, binding of [^18^F]FNDP was blocked with AR-9281 in a dose-dependent manner and approached full occupancy with a dose of 1.0 mg/kg. Blocking was observed in all brain regions studied. The results of high-dose blocking PET-[^18^F]FNDP experiments with AR-9281 (1 mg/kg) and nor-fluoro-FNDP (2 mg/kg, previous report [19]) were nearly comparable.

Because sEH inhibitors can increase peripheral vasodilation and reduce blood pressure [46], affecting cerebral blood flow and radiotracer delivery, the rate constant *K*_1_ estimated from the 2TCM was compared between the baseline and each blocking scan. The estimated *K*_1_ values were slightly increased for all blocking scans as compared to the *K*_1_ estimated from the baseline scan, indicating that reductions in *V*_*T*_ values with blocking were not induced by increased blood flow and tracer delivery.

The efficient blocking of [^18^F]FNDP with two structurally distinct and selective sEH inhibitors, AR-9281 (in this study) and nor-fluoro-FNDP (in our previous study [19]) provides evidence that the radiotracer binding is specific to sEH. The high binding potential (BP_ND_ = 8–16) in the baboon brain suggests that [^18^F]FNDP may be an excellent radiotracer for PET imaging of brain sEH [47].

### [^18^F]FNDP radiometabolite study in plasma and brain

Most PET radiotracers for brain imaging undergo metabolism. Depending on structural properties of the radiometabolites, they may cross the BBB, confounding accurate analysis. Metabolism of xenobiotics normally leads to hydrophilic metabolites *via* oxidation of the parent xenobiotic or coupling with water-soluble groups with poor BBB permeability [48, 49]. Ideally, for clinically viable PET tracers their radiometabolites should enter the brain only minimally (< 5%).

RP-HPLC analysis of radiometabolites in mouse and non-human primate plasma demonstrated that in both species, [^18^F]FNDP forms the same hydrophilic radiometabolites at a similar rate (~ 50% at 30 min post-injection). However, most of the radioactivity in the mouse brain was unchanged parent [^18^F]FNDP (98–99%). Only one radiometabolite (Met-1) entered the mouse brain in a small amount (1.7%), while the concentration of Met-1 in the mouse blood was 10%. This small amount of radiometabolite Met-1 in the mouse brain is probably due, in part, to the presence of residual blood in the cerebral blood vessels. At the end of the PET scan (60–90 min), Met-1 reached a plateau in an amount of 22% in the baboon blood. It is reasonable to hypothesize that at the end of the PET scan, the concentration of Met-1 in the baboon brain will be proportional to its concentration in the blood (2.2-times higher than that in the mouse brain) and will reach a value of ~ 3.7%, while the rest of the brain radioactivity will be parent. Accordingly, we suggest that most radioactivity in the baboon brain is [^18^F]FNDP (> 95%), and mathematical modeling of brain radiometabolites for quantification of the PET scan is unnecessary.

### Radiation dosimetry of [^18^F]FNDP

A major objective of this preclinical study was to perform full-body biodistribution of [^18^F]FNDP in mice and calculate radiation dose estimates for translation of [^18^F]FNDP to human subjects. The study demonstrated that most human organs should receive about 0.01–0.05 mSv/MBq (0.03 to 0.15 rem/mCi). The urinary bladder would receive the highest dose, ~ 0.17 mSv/MBq (0.61 rem/mCi). The effective human dose would be 0.032 mSv/MBq (0.117 rem/mCi). That is similar to other ^18^F-labeled brain agents for human PET.

In the future, it is also important to study the peripheral distribution and pharmacokinetics of [^18^F]FNDP. We have shown that the [^18^F]FNDP is metabolized, and the metabolites are hydrophilic that do not pass BBB. Thus, they will not affect brain imaging. However, those metabolites will have to be considered very carefully with respect to pharmacokinetics relative to the parent when interpreting and quantifying images in the periphery.

## Conclusion

This study demonstrated that [^18^F]FNDP can be used for highly specific in vivo PET imaging of sEH in the baboon brain. Dose-escalation blocking studies with AR-9281, a selective sEH inhibitor that is structurally dissimilar to FNDP, confirmed that uptake of [^18^F]FNDP is specific for sEH. [^18^F]FNDP PET can provide reliable estimates of *V*_*T*_ with a two-tissue-three-compartment model and is suitable for evaluation of sEH inhibitors. In agreement with the high specific binding of sEH, the baboon brain regional distribution of [^18^F]FNDP correlates with regional RNA expression of *EPHX2*, which encodes sEH.

HPLC analysis demonstrated that [^18^F]FNDP forms hydrophilic radiometabolites in mouse and baboon plasma. These radiometabolites of [^18^F]FNDP minimally enter the animal brain, suggesting that their inclusion in PET image analysis is unnecessary. Radiation dosimetry studies in mice showed that the radiation burden of [^18^F]FNDP PET is low and radiotracer is radiologically safe for future human studies.

In summary, [^18^F]FNDP is a safe and specific PET imaging agent for sEH with favorable properties for quantitative imaging in the animal brain. [^18^F]FNDP holds promise for translation to human subjects. PET imaging of sEH with [^18^F]FNDP would provide mechanistic information to facilitate understanding of the neurobiology of various brain diseases.

## Data Availability

Please contact the authors for data request.

## Abbreviations

1TCM: One-tissue-two-compartment
2TCM: Two-tissue-three-compartment
3D: Three dimensional
AD: Alzheimer’s disease
AR-9281: 1-(1-Acetyl-piperidin-4-yl)-3-adamantan-1-yl-urea
BBB: Blood–brain barrier
BP_ND_: Non-displaceable binding potential
EETs: Epoxyeicosatrienoic acids
[^18^F]FNDP: *N*-(3,3-diphenylpropyl)-6-^18^F-fluoronicotinamide
FWHM: Full width at half maximum
HRRT: High-resolution research tomograph
HPLC: High-performance liquid chromatography
MANOVA: Multivariate analysis of variance
PET: Positron emission tomography
sEH: Soluble epoxide hydrolase
TAC: Time–activity curves
VCI: Vascular cognitive impairment
VOI: Volume of interest
*V*_ND_: Distribution volume of non-displaceable radioligand
*V*_*T*_: Distribution volume
